# Online Problem Gambling: A Comparison of Casino Players and Sports Bettors via Predictive Modeling Using Behavioral Tracking Data

**DOI:** 10.1007/s10899-020-09964-z

**Published:** 2020-07-20

**Authors:** Ivan Ukhov, Johan Bjurgert, Michael Auer, Mark D. Griffiths

**Affiliations:** 1 LeoVegas, Stockholm, Sweden; 2 Neccton GmbH, Lienz, Austria; 3 Nottingham Trent University, Nottingham, UK

**Keywords:** Behavioral tracking, Online casino gambling, Online sports betting, Problem gambling, Remote gambling

## Abstract

In this study, the differences in behavior between two groups of online gamblers were investigated. The first group comprised individuals who played casino games, and the second group comprised those who bet on sports events. The focal point of the study was on problem gambling, and the objective was to identify and quantify both common and distinct traits that are characteristic to casino and sports problem gamblers. To this end, a set of gamblers from the gaming operator *LeoVegas* was studied. Each gambler was ascribed two binary variables: one separating casino players from sports bettors, and one indicating whether there was an exclusion related to problem gambling. For each of the four combinations of the two variables, 2500 gamblers were randomly selected for a thorough comparison, resulting in a total of 10,000 participants. The comparison was performed by constructing two predictive models, estimating risk scores using these models, and scrutinizing the risk scores by means of a technique originating from collaborative game theory. The number of cash wagers per active day contributed the most to problem-gambling-related exclusion in the case of sports betting, whereas the volume of money spent contributed the most to this exclusion in the case of casino players. The contribution of the volume of losses per active day was noticeable in the case of both casino players and sports bettors. For casino players, gambling via desktop computers contributed positively to problem-gambling-related exclusion. For sports bettors, it was more concerning when the individual used mobile devices. The number of approved deposits per active day contributed to problem-gambling-related exclusion to a larger extent for sports bettors than casino players. The main conclusion is that the studied explanatory variables contribute differently to problem-gambling-related exclusion among casino players and sports bettors.

## Introduction

The growth of online gambling, driven by broadband penetration and increased market regulation, has brought concerns regarding the impact on gambling habits (Gainsbury 2015). At the same time, in contrast to land-based gambling, online gambling offers possibilities to address these concerns by enabling the collection of rich datasets that can be used in order to attain a better understanding of problem gambling (Philander 2014). This knowledge can subsequently be utilized in order to identify problem gambling at early stages (Sarkar et al. 2016) and to devise adequate strategies for providing protection and support (Auer et al. 2018; van der Maas et al. 2019).

The possibilities of using data collected from individuals engaging in online gambling have been studied and compared to other methods used for collecting data, such as surveys (Griffiths 2014). It has been argued that datasets from online gambling offer a number of advantages for researchers, because they provide an objective account of what gamblers do online (Griffiths 2014).

Using data from *bwin*, Braverman and Shaffer (2012) analyzed the behavior of 530 sports bettors during their first month of activity in terms of intensity, frequency, variability, and trajectory by applying k-means clustering (Hastie et al. 2009). Based on this methodology, a high-risk group was identified, and 70% of the identified individuals were later found to either voluntarily self-exclude or close their accounts. Dragičević et al. (2011) extended this study by incorporating casino players using data from *GTECH G2* and suggested that future work should investigate different gaming segments, extend the set of features, and apply other statistical techniques for prediction, such as logistic regression (Hastie et al. 2009).

Another attempt to identify appropriate methodologies for predicting self-exclusion was posited by Philander (2014). The utility of nine statistical techniques was evaluated on a dataset of sports bettors with the conclusion that artificial neural networks (Hastie et al. 2009) yielded the best performance. However, neural networks are known to be difficult to interpret, and there is generally a trade-off between predictive power and interpretability, which was further explored in the context of responsible gambling by Sarkar et al. (2016).

A paper by Percy et al. (2016) also addressed the problem of predicting individuals who are likely to self-exclude from gambling. The authors applied a set of four statistical methodologies to a dataset from *IGT*. The main finding was that random forest (Hastie et al. 2009) performed best. Additionally, it was suggested that future research should study larger samples in order to obtain a better understanding of how the explanatory variables describing gamblers’ behavior contribute to the model’s performance.

Several authors have studied explanatory variables that are specific to sports bettors and noted the importance of such variables as young age (Abbott et al. 2016), male gender, being single, having impulsive responses to betting opportunities, increased game frequency and expenditure (Hing et al. 2016), proportion of bets made on Saturdays, declined deposits (PricewaterhouseCoopers & Responsible Gaming Council of Canada 2016, 2017), and betting on mobile devices (Lundberg et al. 2018). Russell et al. (2018) reported that placing a high proportion of money on in-play betting, such as betting on the next point in tennis, was related to problem gambling. Other related studies, such as that by LaBrie and Shaffer (2011), made use of data describing online sports betting with the objective of discriminating sports bettors with self-reported problems from sports bettors without such difficulties. In addition, an extensive survey on the topic of sports betting was conducted by Palmer (2014). The survey concluded that sports bettors constituted a clearly unique cohort of gamblers and stressed the need for further studies into sports betting and problem gambling.

It is also important to note that the use of self-exclusion as a proxy for problem gambling—which is the case in many of the above studies—is controversial and has drawn a lot of attention in the literature. Several studies have shown that gamblers with problematic behavior may not self-exclude, while those without problematic behavior may self-exclude for other reasons than problem gambling (Auer and Griffiths 2016; PricewaterhouseCoopers & Responsible Gaming Council of Canada 2017).

To summarize, there are a number of concerns that are commonly raised in the literature. First, there is generally a need for studies into problem gambling in the context of online gambling. The topic is still relatively new and has not been satisfactorily explored. Second, there is a call for comparing different segments of gamblers, since there are large variations in behavior, and scrutinizing and contrasting individual cohorts might shed more light on what drives addiction. Third, the interpretability of modeling techniques generally decreases as their complexity increases. This puts a hard limit on the extent of conclusions drawn from predictions about the relationship between explanatory variables and problem gambling. Finally, there is a need for more representative proxies for problem gambling. An arbitrary type of exclusion from gambling activities might reveal little about problem gambling.

The focal point of the present study was on problem gambling, and the objective was to identify and quantify both common and distinct traits that are characteristic to casino and sports problem gamblers. To do this, a set of gamblers from an online gambling platform was studied by constructing and applying predictive models, evaluating the risk associated with problem-gambling-related exclusion, and subsequently analyzing the outcome by means of collaborative game theory.

## Method

The methodology for studying differences between casino players and sports bettors comprised the following three phases. First, for each group (casino players and sports bettors), a predictive model was trained with the objective of differentiating between individuals who had been excluded due to problem-gambling-related reasons and those who had not been excluded due to problem-gambling-related reasons, by means of a number of demographic and behavioral indicators (defined in the Procedure section below). Second, the contributions of the aforementioned indicators to the final scores were calculated on the level of individual gamblers (defined in the Analysis section below). Third, using these contributions, the inner workings of the two models were compared in order to draw conclusions about the two groups of gamblers with respect to problem-gambling-related exclusion.

### Participants

The online gambling service provider whose data were used for the present study was the gaming operator *LeoVegas*. The extraction of the data was performed in February 2019 and included all relevant historical data available at that moment. The only requirement to an individual for being eligible for the inclusion in the study was a positive approved deposit, which resulted in around 1.2 million accounts. Each eligible gambler was ascribed two binary variables: one indicated whether it was a casino player or a sports bettor, and the other indicated whether the individual had been excluded due to problem-gambling-related reasons (irrespective of when the exclusion had taken place). The decision about the preferred vertical was based on the total amount of actual money wagered. In this regard, there were naturally cases with relatively balanced wagering amounts with respect to casino and sports. However, each gambler was assigned to strictly one group (that is, the one with the largest amount of money wagered). Overall, the proportion of casino players was 87% (therefore, sports bettors constituted 13%), and the proportion of exclusions was around 6% in each group.

Around 70% gamblers (approximately 850,000 accounts) were randomly selected from the pool of eligible gamblers and used for building predictive models, which is discussed in the next section. The remaining 30% of eligible gamblers (approximately 350,000 accounts) were considered for the analysis presented in this paper. More specifically, for each of the four combinations of the two indicator variables mentioned above, 2500 gamblers were randomly selected from the remaining 30% of eligible gamblers, resulting in a total of 10,000 gamblers that were scrutinized.

### Procedure

In regards to modeling gamblers’ behavior from the standpoint of problem gambling, there are two key aspects to address: (i) the target variable and (ii) the explanatory variables. The former is what the model is supposed to predict, and the latter comprise the information that is available at the model’s disposal in order to make predictions. The target variable was problem-gambling-related exclusion, which was defined as follows. Each instance of exclusion was either initiated voluntarily by individuals themselves using the corresponding functionality on the gambling website (self-exclusion) or enforced by the staff due to their own concerns about individuals’ gambling habits (staff-exclusion). Regarding the former, there were license-induced variations in the way the exclusion action was presented to gamblers in the user interface. In some markets, it was made clear that the exclusion is due to problem gambling, and it was permanent. In other markets, it was presented as a long-term exclusion without a permanent option or further details.

In relation to the explanatory variables, after a feature screening and selection process, 40 explanatory variables were chosen for the purposes of the present study. The explanatory variables are listed and described in brief in Table 1. The variables cover a number of demographic aspects, namely age, gender, and country, and a number of behavioral aspects from the beginning until the end of a typical gambler journey, including the number of login sessions, deposits, wagers, and withdrawals.

There were two predictive models constructed: one for casino players and one for sports bettors. Each model was a classifier that was trained to distinguish exclusion cases (referred to as positives) from non-exclusion ones (referred to as negatives). To this end, 70% of eligible gamblers—which contained more than one million accounts, as discussed in the previous section—were chosen randomly and utilized for training.

The modeling technique utilized was gradient boosting (Hastie et al. 2009) and, more specifically, regularized gradient boosting based on decision trees (Chen and Guestrin 2016). A predictive model *f* of this kind has the following additive structure:

1 $$\begin{aligned} {\hat{y}} = f({{{\varvec{x}}}}) = \phi \left( \sum _{i = 1}^n \psi _i({{{\varvec{x}}}})\right) \end{aligned}$$

where ${\hat{y}} \in [0, 1]$ is the prediction (in our case, the risk associated with problem-gambling-related exclusion) for a given set of explanatory variables ${{{\varvec{x}}}} \in {\mathbb {R}}^m$, and *m* is the total number of explanatory variables. Function $\psi _i \in \Psi$, $i = 1, \dots , n$, corresponds to the decision tree constructed during iteration *i* of the training process where $\Psi$ denotes an appropriate space of decision trees, and *n* denotes the total number of iterations. Finally, $\phi (z) = e^z / (1 + e^z)$ is the standard logistic function, coercing the output to the unit interval where zero and one correspond to negative and positive classes, respectively. Decision trees are constructed sequentially in such a way that the objective of each new decision tree is to correct mistakes made by the previous trees (see Chen and Guestrin 2016 for further details).

It is worth noting that a classifier is typically accompanied by a threshold that serves as a decision rule separating negative predictions from the positive ones. For the purposes of this study, there was no need for such a threshold. Raw scores were studied directly, which is elaborated in the next section.

### Analysis

In order to investigate the relationship between the explanatory variables and problem-gambling-related exclusion, the risk scores produced by the predictive models for 10,000 gamblers were analyzed individually. The key aspect to note in this context is that the analysis was not based on the parameters of the models (which remain the same for all possible inputs) but rather on the scores produced by the models for individual gamblers. This type of interpretability of predictive models is known as local, and it allows one to provide a personalized explanation in each particular case.

To elaborate, in order to evaluate the contribution of each explanatory variable to the final risk score and to do so locally, cooperative game theory was utilized and, more specifically, Shapley values (Shapley 1953). Shapley values provide a mechanism for distributing the gain that is obtained by a number of individuals playing a game. In the context of machine learning, a general framework for interpreting predictions by means of Shapley values was developed by Lundberg and Lee (2017), and a fast yet still exact implementation of this approach for the family of predictive models based on tree ensembles was developed by Lundberg et al. (2018). The approach is based on constructing a so-called explanation model *g* for the original model *f* so that

2 $$\begin{aligned} g({{{\varvec{z}}}}) \approx f(h_{{{{\varvec{x}}}}}({{{\varvec{z}}}})) \end{aligned}$$

whenever ${{{\varvec{z}}}} \approx {{{\varvec{x}}}}$. Here *h* is an auxiliary mapping that allows the explanation model to operate on a simplified set of variables ${{{\varvec{z}}}} \in \{0, 1\}^m$. The decomposition of a risk score into individual contributions of the explanatory variables takes the following additive form:

3 $$\begin{aligned} g({{{\varvec{z}}}}) = \gamma _0 + \sum _{i = 1}^m \gamma _i \, z_i \end{aligned}$$

where coefficient $\gamma _i$ corresponds to the contribution of explanatory variable *i*. These coefficients are referred to as Shapley values, and they are computed as follows:

4 $$\begin{aligned} \gamma _i(f, {{{\varvec{x}}}}) = \sum _{{{{\varvec{z}}}}} \frac{|{{{\varvec{z}}}}|! \, (m - |{{{\varvec{z}}}}| - 1)!}{m!} (f(h_{{{{\varvec{x}}}}}({{{\varvec{z}}}})) - f(h_{{{{\varvec{x}}}}}({{{\varvec{z}}}} \backslash i))) \end{aligned}$$

where $|\cdot |$ stands for the $L^1$ norm, $\cdot \backslash i$ denotes setting entry *i* to zero, and the summation goes roughly over all possible ${{{\varvec{z}}}}$. It can be seen that the Shapley value of a feature is a weighted average of all possible variations in the output of the model when the feature becomes available at the model’s disposal. It can be demonstrated that the above construction possesses three properties that are highly desirable in the context of distributing contribution: local accuracy, missingness, and consistency (see Lundberg and Lee 2017 for further details).

The analysis presented in this paper was then based on comparing contributions $\{ \gamma _i \}_{i = 1}^m$ of the explanatory variables to the risk of exclusion due to problem gambling that were computed for 10,000 gamblers where 5000 were casino players, and 5000 were sports bettors.

## Results

This section presents the main results. First, casino players and sports bettors are compared by inspecting Shapley values in isolation, meaning that the values of the explanatory variables are not considered at this first step. Following this, the Shapley values are scrutinized in relation to the values of the explanatory variables. The results are further discussed in the next section where the most important findings are emphasized and elaborated on.

Before reporting the Shapley values, some comment on the performance of the trained models is needed. The area under the receiver operating characteristic curve was found to be 0.87 for the casino-gambling model and 0.92 for the sports-betting model. However, the imbalance of the data has to be taken into consideration when interpreting these figures (given that the proportion of positive examples was around 6%). In such cases, the precision and recall metrics are usually preferred. These metrics require converting estimated risk scores, which are values from zero to one, into binary decisions. As mentioned in the Procedure section, this was not necessary for the analysis presented in this paper, since it operated directly on raw risk scores. Nonetheless, for completeness, two thresholds were chosen, one for each mode, by optimizing the F score with $\beta = 0.5$. The precision and recall were found to be 0.45 and 0.27, respectively, for the casino-gambling model and 0.60 and 0.42, respectively, for the sports-betting model.

### Aggregate Contributions

**Fig. 1:**
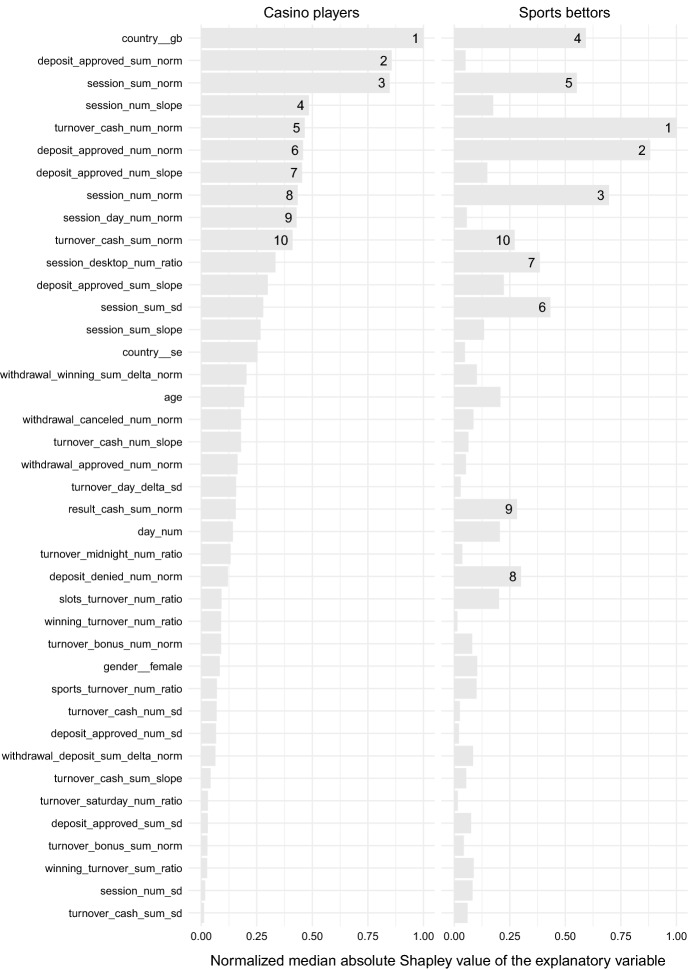
Contribution magnitudes. The explanatory variables are sorted with respect to the casino-gambling group

Figure 1 shows the contributions of the 40 explanatory variables to the risk associated with problem-gambling-related exclusion (refer to Table 1 for the meaning of the variables). The impact was measured in terms of the median absolute value of Shapley values, which was further normalized for convenience. The top ten indicators for each group of gamblers are labeled in the figure.

Comparing the two groups, it can be seen that there are significant differences in terms of which variables are important. Only six out of ten major contributors in the case of casino players can be found in the top ten of sports bettors. More specifically, the slope of the number of approved deposits denoted by deposit_approved_num_slope, volume of approved deposits denoted by deposit_approved_sum_norm, number of active days denoted by session_day_num_norm, and slope of the number of sessions denoted by session_num_slope are less informative for sports betting compared to casino gambling. Likewise, the number of denied deposits denoted by deposit_denied_num_norm, volume of cash (as opposed to bonus) results denoted by result_cash_sum_norm, proportion of desktop authentication sessions denoted by session_desktop_num_ratio, and standard deviation of the duration of sessions denoted by session_sum_sd are less informative for casino gamblers compared to sports bettors.

The variable indicating that the account was registered in the United Kingdom, which is denoted by country__gb, stands out among other explanatory variables. This is due to the fact that the exclusion rate is significantly higher in the market compared to other markets of operation. It is also interesting to note that the other demographic variables, namely age and gender, play a role, but this role is relatively small compared to other indicators. It suggests that, when problem-gambling-related exclusion is concerned, age and gender are not as informative as one might expect.

**Fig. 2:**
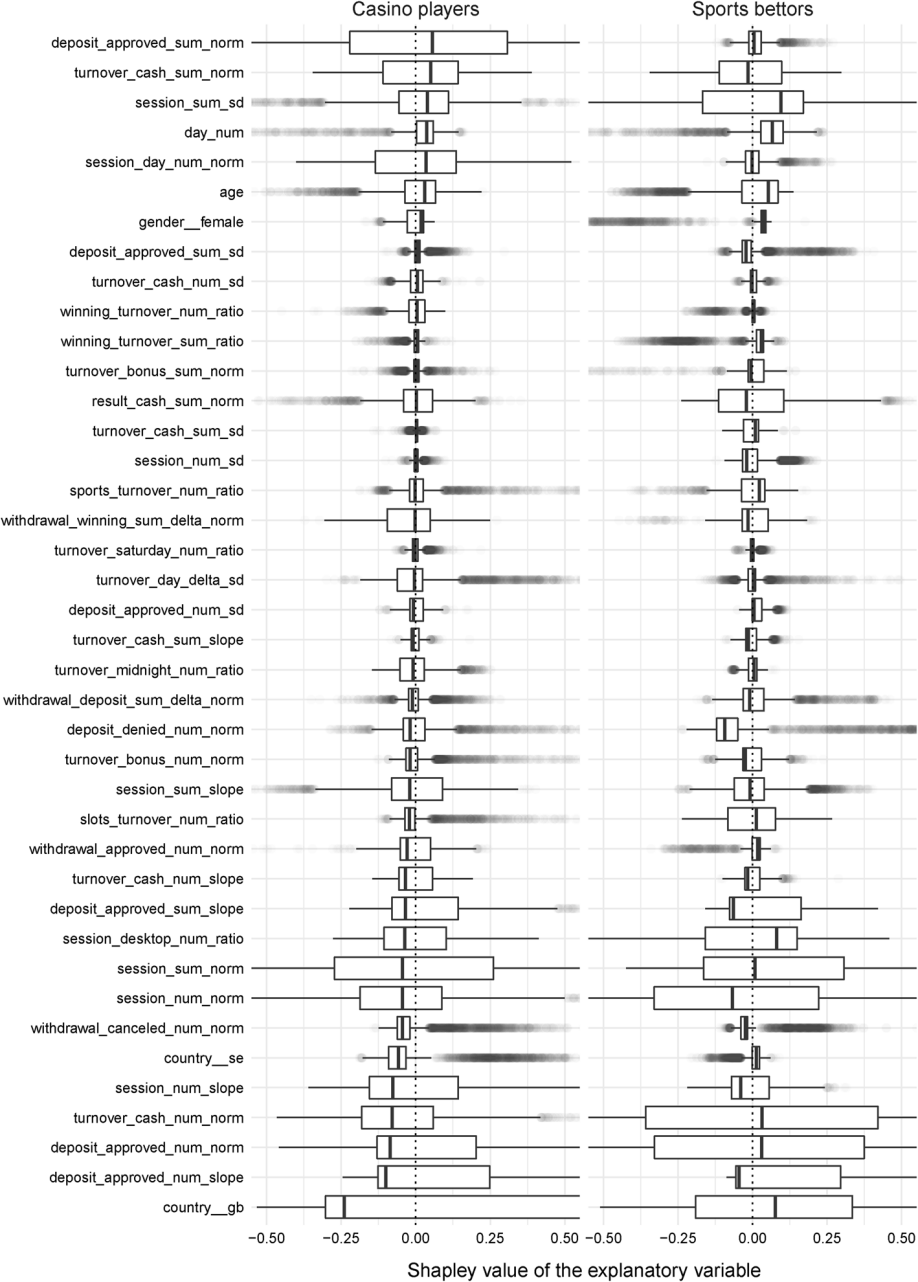
Contribution distributions. The variables are sorted by the median Shapley value

Figure 2 shows box plots of Shapley values of all explanatory variables. Outliers are depicted by semi-transparent circles. The variables are sorted by their median Shapley values in the casino-gambling group, and the graph is zoomed in on the interquartile ranges for clarity reasons. It can be seen that the distributions tend to be skewed toward zero. More specifically, the variables with negative medians are right skewed, while those with positive medians are left skewed. One can also note that the interquartile ranges of relatively few variables are located strictly to the left or right of zero. Examples of such variables include the number of days since registration denoted by day_num, which mainly increases the risk score for both groups, and the number of canceled withdrawals denoted by withdrawal_canceled_num_norm, which mainly decreases the risk score for both groups.

The distribution of the Shapley values of deposit_approved_sum_norm is much more spread out for casino players. This means that the variable’s contribution to the risk score varies substantially, taking relatively large negative and positive values. For sports bettors, this is not the case. Here the variable has a very narrow range of contribution. A similar observation can be made with respect to session_daynum_norm (the number of active days). On the other hand, the contribution of the number of cash wagers denoted by turnover_cash_num_norm is relatively similar across the two groups, which can also be concluded with respect to self-reported age.

The risk score is noticeably indifferent to specific variables. For casino players, the standard deviation of the volume of approved deposits denoted by deposit_approved_sum_sd, ratio of the volume of cash winnings to the volume of cash wagers denoted by winning_turnover_sum_ratio, volume of bonus wagers denoted by turnover_bonus_sum_norm, standard deviation of the volume of cash wagers denoted by turnover_cash_sum_sd, standard deviation of the number of authentication sessions denoted by sessions_num_sd, and proportion of cash wagers on Saturdays denoted by turnover_saturday_num_ratio are tightly centered at zero. For sports bettors, such variables are the standard deviation of the number of cash wagers denoted by turnover_cash_num_sd and ratio of cash winnings to cash wagers denoted by winning_turnover_num_ratio. The impact of these variables on the score in the corresponding groups was observed to be minor.

Overall, there were both similarities and dissimilarities between casino players and sports bettors.

### Individual Contributions

In this section, the top ten casino-gambling and the top ten sports-betting variables as identified in the previous section are examined. Age is also added to the list, as this is usually of interest. Consequently, the variables of interest comprise the following 15 indicators: age, country__gb (and country__se), deposit_approved_num_norm (the number of approved deposits), deposit_approved_num_slope, deposit_approved_sum_norm, deposit_denied_num_norm, result_cash_sum_norm, session_day_num_norm, session_desktop_num_ratio, session_num_norm (the number of authentication sessions), session_num_slope, session_sum_norm (the duration of authentication sessions), session_sum_sd, turnover_cash_num_norm, and turnover_cash_sum_norm (the number of cash wagers). Unlike the previous section, the Shapley values in this section are shown in relation to the individual values of the corresponding explanatory variables.

In the majority of the figures that follow, the overall trend is emphasized by a solid line computed using locally estimated scatterplot smoothing (Hastie et al. 2009), and the border between negative and positive Shapley values is highlighted using a dashed line. In addition, many plots have logarithmic scales on their horizontal axes with values of interest being annotated.

#### Effect of the Country of Registration

**Fig. 3:**
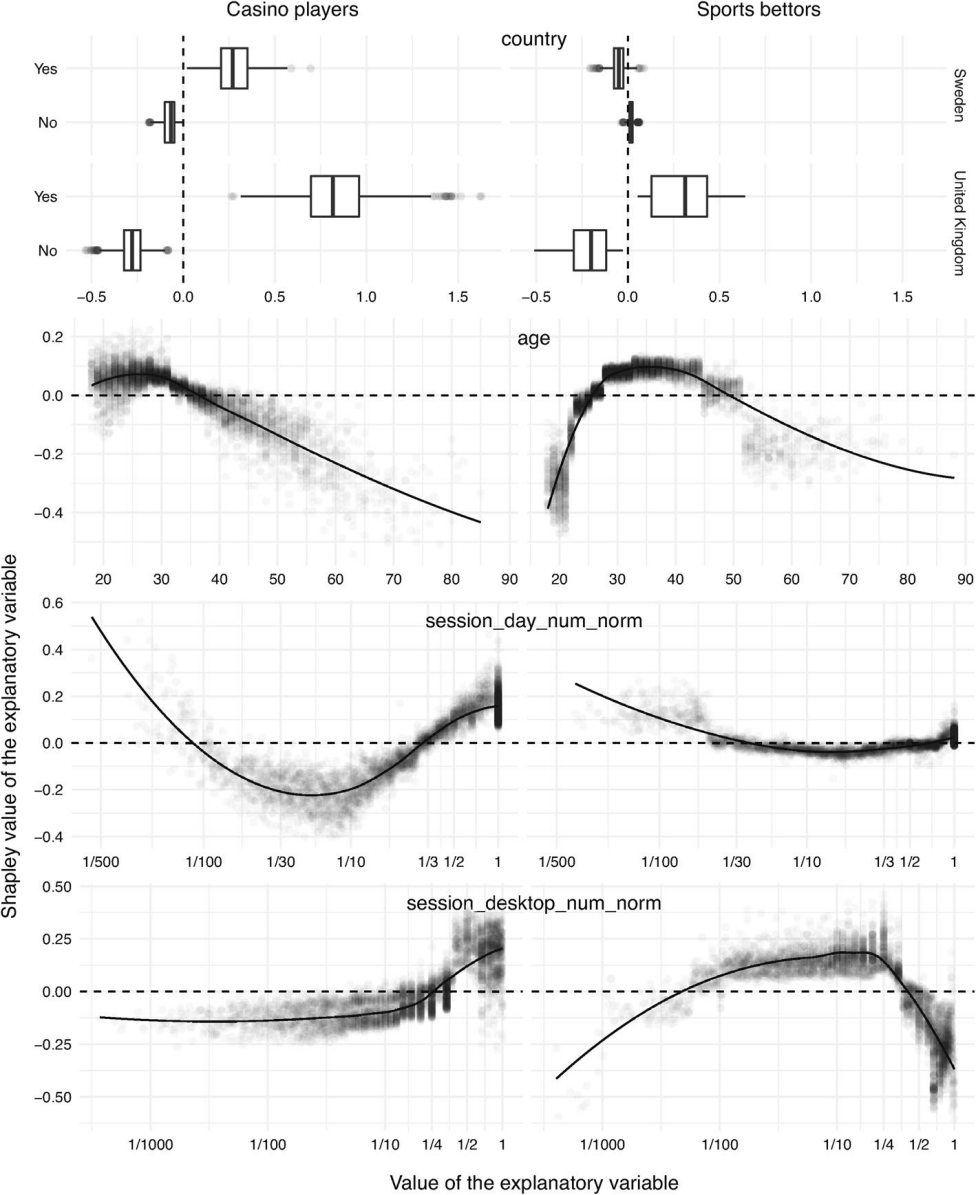
Contribution of demographic and session-related explanatory variables, namely the country of registration (*first*), self-reported age (*second*), active days per day since registration (*third*), and proportion of sessions started on desktop computers (*fourth*)

The first explanatory variable analyzed is the country of registration. There were two binary variables considered: country__gb indicating whether the account was created in the United Kingdom and country__se indicating whether the account was created in Sweden. However, it should be noted that the dataset being studied was not constrained to just these two countries. For other countries, both binary variables were zero. The first row in Fig. 3 shows box plots of Shapley values for the two values of the aforementioned two binary variables. The UK market stands out in terms of the contribution magnitude, which was explained earlier. Focusing closer on the UK indicator (the bottom four box plots), the situation is similar across the two groups of bettors when the variable is zero (that is, not registered in the UK). However, when the variable is one, it manifests itself much stronger in the case of casino players. More specifically, the bulk of the distribution is above 0.5, while it is below 0.5 in the case of sports bettors. This suggests that British casino players are more prone to exclusion than British sports bettors. As for the indicator for Sweden, when the variable is one (that is, registered in Sweden), the risk score is strictly increased for casino players but mostly decreased (although relatively little) for sports bettors. This suggests that Swedish sports bettors tend to not exclude due to gambling-related problems.

#### Effect of Self-reported Age

The second row in Fig. 3 corresponds to the age that was reported by the gambler at initial registration. It can be seen that the two groups have similar patterns. Low and high values tend to decrease the risk score, while the ones in the middle tend to increase. However, for casino players, this middle region is narrower and has a larger vertical spread, and the extremum is reached much earlier. For casino players, the most susceptible age for problem-gambling-related exclusion is between 25 and 30 years, whereas for sports bettors, it is between 30 and 40 years.

#### Effect of Authentication Sessions

The influence of the number of days with authentication sessions, which are also referred to as active days, normalized by the total number of days since registration (that is, session_day_num_norm) is depicted in the third row in Fig. 3. One should be careful reading this plot, since a lot of mass is concentrated at value one, which is due to a large number of new gamblers who have one active day and one day in total. There are differences between casino players and sports bettors. More specifically, the change from negative to positive Shapley values for casino players is one active day per three days. However, there is no clear-cut change point for sports bettors. One can observe that the values in the left tail also tend to increase the risk score. This left tail corresponds to infrequent gamblers with relatively long lifetimes (that is, the time since the initial registration). Such gamblers might decide to permanently close their accounts as redundant, making the model increase the risk score for a reason other than problem gambling.

The proportion of sessions started on a desktop computer including laptops (that is, session_desktop_num_ratio) is depicted in the last row in Fig. 3. A sharp separation can be observed. For casino players, the ratio tends to increase the risk score when it increases to one-quarter or more. For sports bettors, there is an opposite trend. The score starts to decrease as the ratio reaches around one-half. This means that, for casino players, using primarily desktop computers for gambling increases the risk of exclusion, while this mode of gambling decreases the risk for sports bettors.

**Fig. 4:**
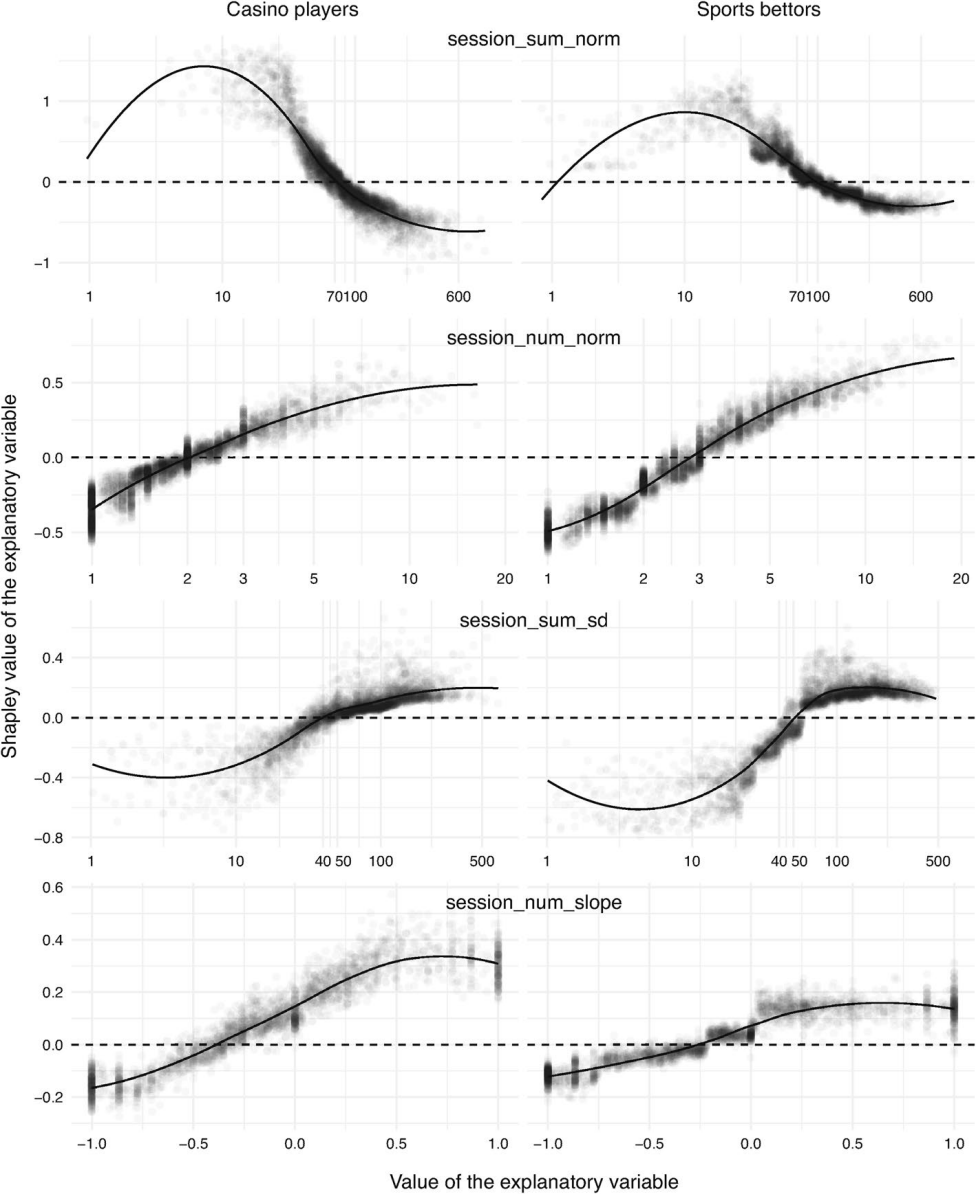
Contribution of session-related variables, namely the duration of sessions per active day in minutes (*first*), number of sessions per active day (*second*), variability in the duration of sessions (*third*) in minute, and slope of the number of sessions (*fourth*)

The impact of the duration of sessions per active day (that is, session_sum_norm) is displayed in the first row in Fig. 4. There is a sharp separation in both groups. However, the change of the sign of Shapley values happens at different times. It is around 70 min for casino players and 100 min for sports bettors. The overall trend declines, which likely relates to the degree of gamblers’ engagement with the product. Gamblers who are willing to spend more time are less inclined to exclusion. This, in turn, might again hint at the limitations of the target variable.

The number of sessions per active day (that is, session_num_norm) is depicted in the second row in Fig. 4. The trend is as expected here. The risk score increases with the frequency of sessions. As with the previous plot, the change point is slightly different for the two groups. It is around two sessions per day for casino players and three sessions per day for sports bettors.

The utility of the standard deviation of the duration of sessions (that is, session_sum_sd) is illustrated in the third row in Fig. 4. This explanatory variable was available for 84% of the 10,000 gamblers. It can be seen that the variable is informative, and that it manifests itself similarly but noticeably stronger among sports bettors in the negative region of Shapley values.

The correlation coefficient of the slope of the number of sessions per active day over the latest three months (that is, session_num_slope) is given in the fourth row in Fig. 4. The figure concerns around 65% of the gamblers. The transition of Shapley values from negative to positive happens at different locations: −0.4 for casino players and −0.25 for sports bettors. The trend on the positive half-line is noticeably flatter for sports bettors. In other words, the contribution to the risk score for sports bettors plateaus at a specific point, while it keeps growing for casino players.

#### Effect of Approved and Denied Deposits

**Fig. 5:**
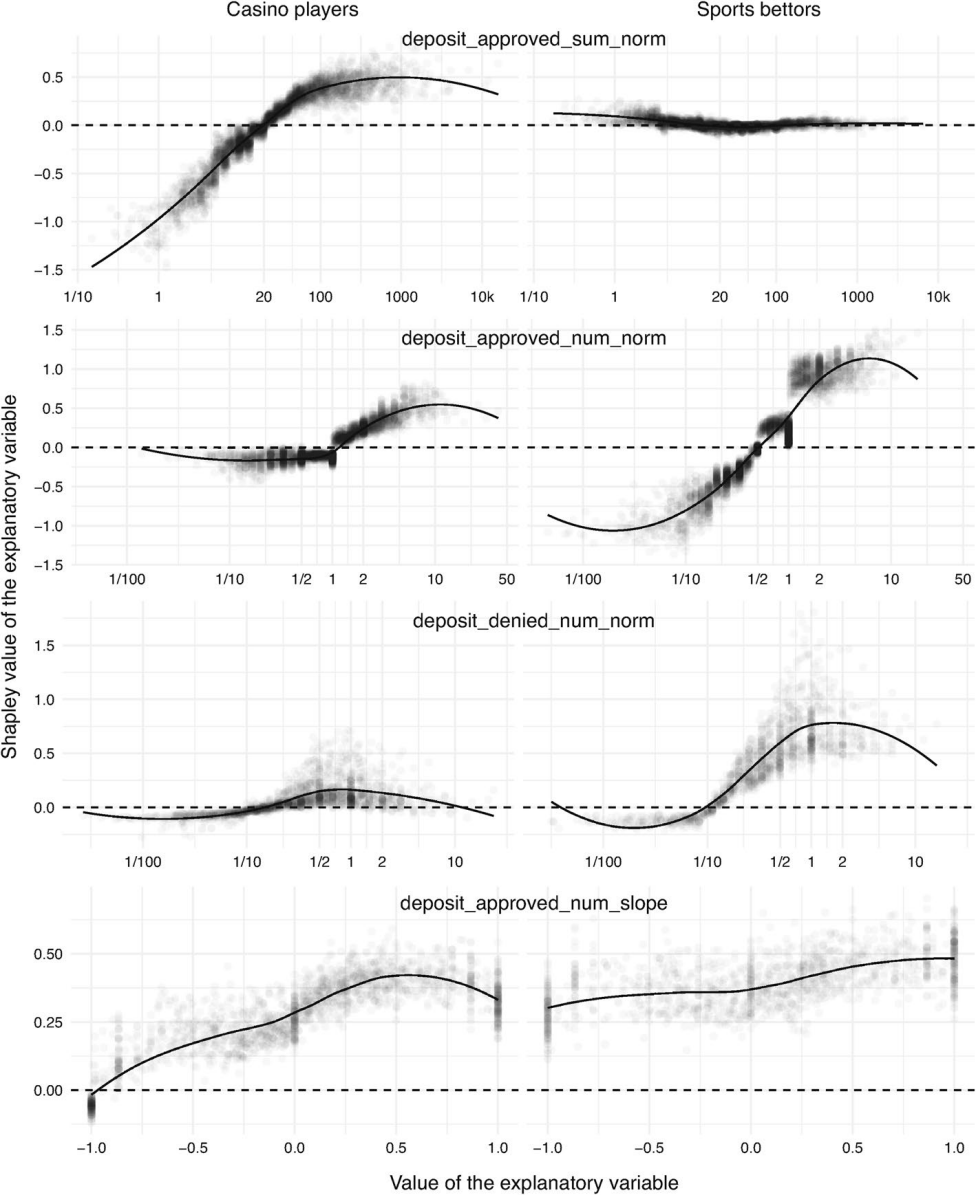
Contribution of deposit-related explanatory variables, namely the volume of approved deposits per active day (*first*), number of approved deposits per active day (*second*), number of denied deposits per active day (*third*), and slope of the number of approved deposits (*fourth*). All financial quantities are in euros

In relation to depositing behavior, the volume of approved deposits per active day (that is, deposit_approved_sum_norm) is depicted in the first row in Fig. 5. The casino-gambling group has a large spread of Shapley values, indicating high informativeness of the variable in this case. For casino players, there is also a clear change point at around €20. A deposit above €20 per active day raises a concern. However, the situation is not as clear for sports bettors. Relative to casino players, the spread of Shapley values appears to be minimal. For the majority of sports bettors, which are located in the middle, the Shapley values fluctuate around zero, meaning that this explanatory variable is not indicative of problem-gambling-related exclusion in the case of sports bettors.

The number of approved deposits per active day (that is, deposit_approved_num_norm) is given in the second row in Fig. 5. In both casino-gambling and sports-betting groups, there is a clear separation between positive and negative Shapley values. For casino players, the critical point is located at one deposit per active day, while it is at one deposit per two active days for sports bettors. In addition, for casino players, the left-hand side is notably flat, meaning that fewer than one approved deposit per active day decreases the risk by a relatively constant amount (independent of the value of the explanatory variable). Finally, sports bettors exhibit another notable change at one deposit per active day; after this point, the contribution exhibits a large jump.

The impact of the number of deposits denied per active day (that is, deposit_denied_num_norm) is shown in the third row in Fig. 5. Denied deposits are due to payment service providers, and they can occur due to various reasons, such as insufficient funds. In this figure, gamblers without denied deposits are excluded for clarity reasons. The behavior appears to be similar across casino players and sports bettors in terms of the change point and dissimilar in terms of the vertical spread, which is similar to the previous observations.

The correlation coefficient of the number of approved deposits per active day over the most recent three months (that is, deposit_approved_num_slope) is depicted in the last row in Fig. 5. It should be noted that this variable is available for around 35% of the participants. The explanatory variable gives an offset to the risk score that is almost exclusively positive. However, the magnitude of this offset tends to be higher to the right of the origin. This trend is particularly prominent for casino players. Also of note is the fact that the scores of casino players take on values from around zero to 0.5, whereas those of sports bettors lie mostly between 0.25 and 0.5, meaning that the variable increases the risk of problem-gambling-related exclusion for sports bettors much more than casino players.

#### Effect of Cash Wagers

**Fig. 6:**
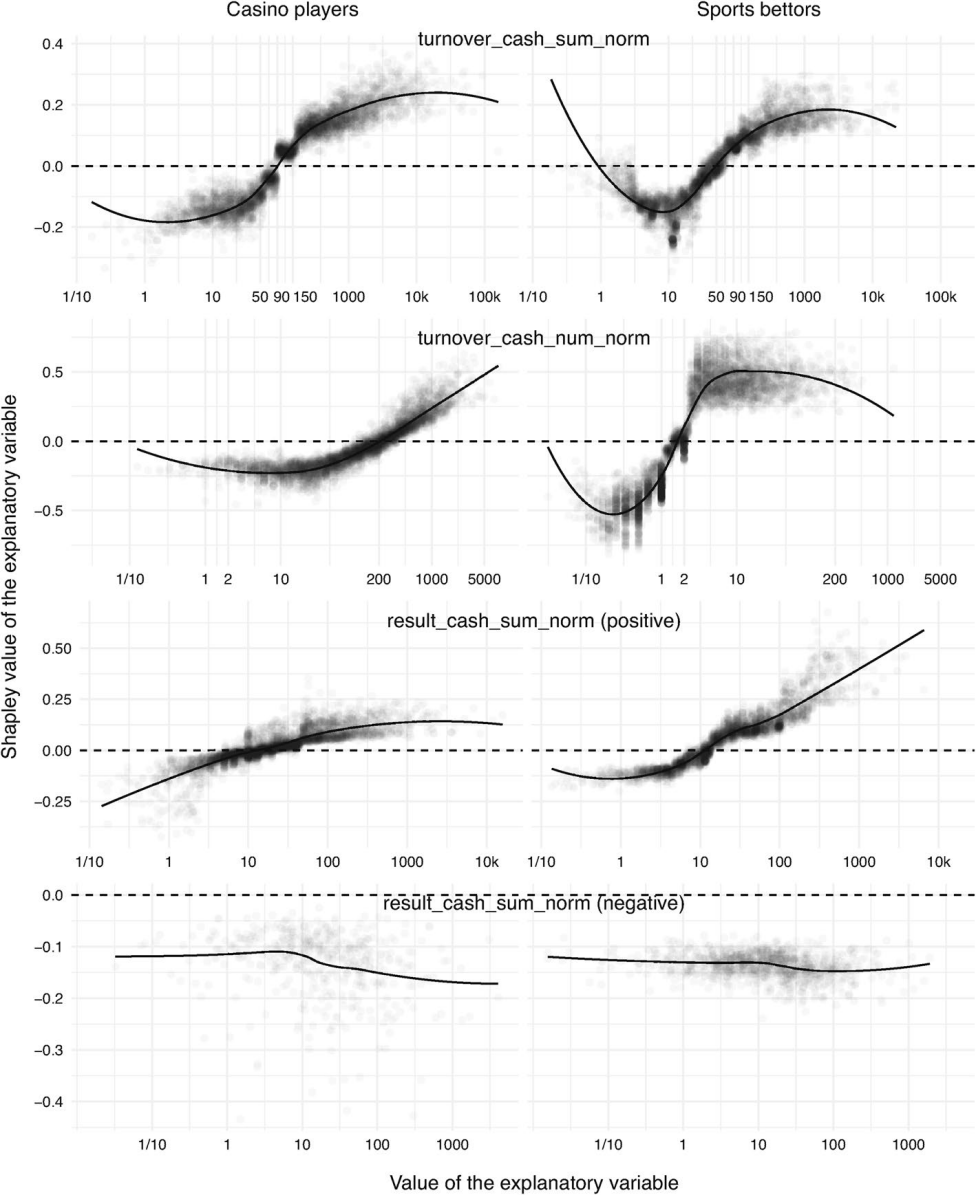
Contribution of wager- and result-related explanatory variables, namely the volume of turnovers per active day (*first*), number of turnovers per active day (*second*), and volume of positive and negative results per active day (*third* and *fourth*). All financial quantities are in euros

The volume of cash wagers per active day (that is, turnover_cash_sum_norm), which refers to real money as opposed to bonus money, is displayed in the first row in Fig. 6. The change point for casino players is €90 per active day but only around €50 for sports bettors. In other words, for sports bettors, the risk of problem-gambling-related exclusion starts to be increased by this explanatory variable at a wager that is €40 lower compared to casino players.

The number of cash wagers per active day (that is, turnover_cash_num_norm) is given in the second row in Fig. 6. Here the difference is dramatic. The critical point is 200 bets per active day for casino players and only two for sports bettors. The difference is explained by the nature of the two types of gambling. A casino player generates a wager with every spin of a slot machine. However, each wager is typically of a small monetary value compared to bets in sports. In addition, it should be noted that the spread of Shapley values in the sports-betting group is larger, indicating that this explanatory variable is more discriminative in the case of sports bettors.

#### Effect of Cash Results

The final variable under examination is the volume of cash results per active day (that is, result_cash_sum_norm), which is the difference between the volume of cash wagers and winnings. Positive and negative results are presented separately. The third row in Fig. 6 shows positive results (in favor of the operator). In general, high losses increase the risk level of problem-gambling-related exclusion. The sign of Shapley values changes at a loss of €10, meaning that after this amount, the risk score starts to be increased by this variable. The last row in Fig. 6 shows negative results (in favor of the gambler). In this case, the small number of data points should be noted when interpreting the results. The Shapley values are all negative, suggesting that gamblers who win tend to not be excluded.

## Discussion

The results demonstrate that the explanatory variables being considered contribute differently to exclusion due to problem-gambling-related reasons among casino players and sports bettors.

It was found that, among the explanatory variables considered, the number of cash wagers per active day contributed the most to problem-gambling-related exclusion in the case of sports betting. Similarly, Hing et al. (2016) found that the risk of problem gambling increased with the frequency of sports betting. However, this variable does not contribute to the same extent in the case of casino players, which can be explained by multitudes of casino spins compared to meager numbers of targeted bets in sports betting. For casino players, the title of the most informative feature was shared between the volume of approved deposits and the duration of authentication sessions, excluding the country of registration due to the reasons mentioned earlier (see the three most influential explanatory variables in Fig. 1).

Age plays a similar but still noticeably different role for casino players compared to sports bettors. In the case of the casino players, the age between 18 and 35 years is associated with large positive Shapley values, increasing the risk score, whereas the critical age for sports bettors is found between 25 and 45 years. Young sports bettors appear to be particularly averse to problem-gambling-related exclusion, which can be seen in Fig. 3. Several other studies, such as Abbott et al. (2016) and Hing et al. (2016), have found age to be an important factor related to problem gambling. However, in the present study, this explanatory variable was dominated by other indicators.

According to the analysis, the number of days with any activity on the gambling website per week was a much stronger informer about problem-gambling-related exclusion for casino players than sports bettors, which can be observed in the second illustration in Fig. 3. The duration and frequency of individual sessions were also important. For casino players, the contributions of the corresponding variables to the risk score change direction at a smaller duration of sessions and at a smaller number of sessions compared to sports bettors.

Similar to the study by Lopez-Gonzalez et al. (2018), the types of devices used to gamble were also found to matter in relation to problem gambling. Moreover, the two groups had opposite trends with respect to desktop versus mobile. For casino players, gambling via desktop computers contributed positively to (that is, increased) problem-gambling-related exclusion. For sports bettors, it was more concerning when the individual used mobile devices.

The number of approved deposits per active day contributed to problem-gambling-related exclusion to a larger extent for sports bettors than casino players. One potential explanation lies in the nature of these two verticals. The number of sports events (and consequently the amount of the corresponding betting opportunities) is limited, while an individual can play casino games continuously. Therefore, an excessive number of deposits might be more concerning for sports bettors, since it might stand out as disproportionate to the betting capacity of the sports vertical. On the other hand, for casino players, the volume of money deposited had a greater impact on the risk score. Here one might reason in a similar way (that is, individual casino wagers are typically small and, therefore, do not require large funds to be available in order to engage in a sufficiently fulfilling gaming experience).

The volume of losses per active day (that is, the positive dimension of result_cash_sum_norm) noticeably contributed to problem-gambling-related exclusion for both casino players and sports bettors, and this contribution was found to grow with the amount of money lost. However, the volume of winnings per active day (that is, the negative dimension of result_cash_sum_norm) did not have a definite reversed trend. This hints at the well-known disproportionate sensitivity of individuals to losing and winning, meaning that gamblers might suffer from a loss more than enjoy a gain of the same financial magnitude (Kahneman and Tversky 1979).

The present study makes a number of major contributions to the gambling literature. The paper provided a structured analysis and comparison of a large set of gamblers that had been active in casino and sports gambling. The analysis focused on explaining how various demographic and behavioral indicators contributed to problem-gambling-related exclusion by applying concepts from machine learning and game theory. In particular, the contributions of 40 explanatory variables were analyzed by means of Shapley values which, despite their introduction more than half a century ago, are considered to be a novel approach due to the latest developments that made this behavioral analysis possible. The present study is also the first to include denied deposits as an indicator of problem gambling, and this indicator was shown to be informative. Denied deposits could be related to insufficient funds, which is a prominent indicator of problem gambling (American Psychiatric Association 2013).

The results could potentially be used to foresee harmful patterns in behavior and take proactive actions by gambling operators. For instance, high deposit amounts and frequencies could be prevented through the use of deposit limits (Auer and Griffiths 2013). Apart from deposit limits, operators can also offer wagering, loss, or time limits; Bonello and Griffiths (2017) found that the majority of operators had at least one of those limits. Another potential mechanism for prevention is reality checks via pop-up messages (Auer and Griffiths 2015; Auer et al. 2014; Stewart and Wohl 2013).

The limitations of the present study originate mainly from the data that the study was based on. First and foremost, even though the study scrutinized exclusion related to problem gambling, the target variable was not problem gambling itself. There were likely to be some cases where gamblers excluded themselves because of reasons other than gambling problems. In addition, there were potentially cases where gamblers utilized multiple platforms or shared accounts, rendering the relevant information on these individuals incomplete. Furthermore, the way the gamblers were split into the two groups (casino players and sports bettors) could also constitute a potential source of errors, since individuals were not restricted to any particular vertical and could engage in both types of gambling activities.

In regard to future work, further studies should focus on connecting the identification of problem gambling with proactive assistance in attaining and maintaining sustainable (that is, responsible) gambling habits. In this context, one might leverage the knowledge about similarities and dissimilarities between casino players and sports bettors in order to treat each group of gamblers in the most appropriate way.

**Table 1:** Explanatory variables used for modeling problem-gambling-related exclusion

	Name	Meaning
1	age	Age
2	country__gb	Indicator if registered in the UK
3	country__se	Indicator if registered in Sweden
4	day_num	Number of days since registration
5	deposit_approved_num_norm	Number of approved deposits
6	deposit_approved_num_sd	Variation in the number of deposits
7	deposit_approved_num_slope	Slope of the number of deposits
8	deposit_approved_sum_norm	Volume of approved deposits
9	deposit_approved_sum_sd	Variation of the volume of deposits
10	deposit_approved_sum_slope	Slope of the volume of deposits
11	deposit_denied_num_norm	Number of denied deposits
12	gender__female	Indicator if female
13	result_cash_sum_norm	Volume of cash wagers minus winnings
14	session_day_num_norm	Number of active days
15	session_desktop_num_ratio	Proportion of desktop sessions
16	session_num_norm	Number of sessions
17	session_num_sd	Variation in the number of sessions
18	session_num_slope	Slope of the number of sessions
19	session_sum_norm	Duration of sessions
20	session_sum_sd	Variation in the duration of sessions
21	session_sum_slope	Slope of the duration of sessions
22	slots_turnover_num_ratio	Proportion of bets on slot machines
23	sports_turnover_num_ratio	Proportion of bets on sports events
24	turnover_bonus_num_norm	Number of bonus wagers
25	turnover_bonus_sum_norm	Volume of bonus wagers
26	turnover_cash_num_norm	Number of cash wagers
27	turnover_cash_num_sd	Variation in the number of cash wagers
28	turnover_cash_num_slope	Slope of the number of cash wagers
29	turnover_cash_sum_norm	Volume of cash wagers
30	turnover_cash_sum_sd	Variation in the volume of cash wagers
31	turnover_cash_sum_slope	Slope of the volume of cash wagers
32	turnover_day_delta_sd	Variation in days between wagers
33	turnover_midnight_num_ratio	Proportion of wagers late at night
34	turnover_saturday_num_ratio	Proportion of wagers on Saturdays
35	winning_turnover_num_ratio	Number of winnings to wagers
36	winning_turnover_sum_ratio	Volume of winnings to wagers
37	withdrawal_approved_num_norm	Number of approved withdrawals
38	withdrawal_canceled_num_norm	Number of canceled withdrawals
39	withdrawal_deposit_sum_delta_norm	Volume of withdrawals minus deposits
40	withdrawal_winning_sum_delta_norm	Volume of withdrawals minus winnings

## Appendix

### Explanatory Variables

The explanatory variables used in the present study and their meaning can be seen in Table 1. The _norm suffix indicates that the corresponding variable was normalized by the number of active days (that is, session_day_num_norm), which were days with at least one authentication session. The only exception was session_day_num_norm where the normalization was done with respect to the number of days since registration (that is, day_num). The _sd suffix stands for *standard deviation*, and variables with this suffix operate on daily aggregates and not on individual events. The _slope suffix refers to Pearson’s correlation coefficient of the regression line computed over a 90-day window (daily aggregates) prior to the last-seen date where the horizontal axis enumerates days with any activity with respect to the explanatory variable in question. Finally, the _ratio suffix indicates a quotient of two quantities, and the _delta suffix indicate a difference between two quantities.

The United Kingdom and Sweden were ascribed individual binary variables (that is, country__gb and country__se, respectively). The choice was mainly due to the fact that the two countries were the largest markets of the gambling platform whose data were used for this study.

The result_cash_sum_norm variable corresponds to the difference between cash wagers and cash winnings (also normalized as described above). In this case, positive values are in favor of the gambling service provider, while negative values are in favor for the gambler.

The session_desktop_num_ratio variable was computed by dividing the number of authentication sessions made from desktop computers including laptops by the total number of sessions.

The turnover_midnight_num_ratio variable was computed by dividing the number of cash wagers made from midnight until four o’clock in the morning by the total number of cash wagers.
